# CSH guidelines for the diagnosis and treatment of drug-induced liver injury

**DOI:** 10.1007/s12072-017-9793-2

**Published:** 2017-04-12

**Authors:** Yue-cheng Yu, Yi-min Mao, Cheng-wei Chen, Jin-jun Chen, Jun Chen, Wen-ming Cong, Yang Ding, Zhong-ping Duan, Qing-chun Fu, Xiao-yan Guo, Peng Hu, Xi-qi Hu, Ji-dong Jia, Rong-tao Lai, Dong-liang Li, Ying-xia Liu, Lun-gen Lu, Shi-wu Ma, Xiong Ma, Yue-min Nan, Hong Ren, Tao Shen, Hao Wang, Ji-yao Wang, Tai-ling Wang, Xiao-jin Wang, Lai Wei, Qing Xie, Wen Xie, Chang-qing Yang, Dong-liang Yang, Yan-yan Yu, Min-de Zeng, Li Zhang, Xin-yan Zhao, Hui Zhuang, Drug-induced Liver Injury (DILI) Study Group, Chinese Society of Hepatology (CSH), Chinese Medical Association (CMA)

**Affiliations:** 1 Liver Disease Center of PLA, Bayi Hospital, Nanjing University of Chinese Medicine, Nanjing, 210002 China; 2 Department of Gastroenterology, Renji Hospital, School of Medicine, Shanghai Jiaotong University, Shanghai, 200001 China; 3 Shanghai Liver Diseases Research Center, 85th Hospital, Nanjing Military Command, Shanghai, 200235 China; 4 Hepatology Unit, Department of Infectious Diseases, Nanfang Hospital, Southern Medical University, Guangzhou, 510515 China; 5 Liver Diseases Center, Second Xiangya Hospital, Central South University, Changsha, 410011 China; 6 Department of Pathology, Eastern Hepatobiliary Surgery Hospital, Second Military Medical University, Shanghai, 201805 China; 7 Department of Infectious Disease, Shengjing Hospital of China Medical University, Shenyang, 110004 China; 8 Artificial Liver Center, Beijing Youan Hospital, Capital Medical University, Beijing, 100069 China; 9 Department of Gastroenterology, Second Affiliated Hospital, Xi’an Jiaotong University, Xian, 710004 China; 10 Department of Infectious Diseases, Institute for Viral Hepatitis, Second Affiliated Hospital, Chongqing Medical University, Chongqing, 400010 China; 11 Department of Pathology, School of Medicine, Fudan University, Shanghai, 200433 China; 12 Liver Research Center, Beijing Friendship Hospital, Capital Medial University, Beijing, 100069 China; 13 Department of Infectious Diseases, Ruijin Hospital, School of Medicine, Shanghai Jiaotong University, Shanghai, 200025 China; 14 Department of Hepatobiliary Disease, Fuzhou General Hospital of PLA, Fuzhou, 350025 China; 15 Department of Liver Disease, Shenzhen Third People’s Hospital, Shenzhen, 518040 China; 16 Department of Gastroenterology, Shanghai First People’s Hospital, School of Medicine, Shanghai Jiaotong University, Shanghai, 200080 China; 17 Department of Infectious Diseases, Kunming General Hospital of PLA, Kunming, 650032 China; 18 Department of Traditional and Western Medical Hepatology, Third Affiliated Hospital, Hebei Medical University, Shijiazhuang, 050051 China; 19 Department of Microbiology and Infectious Disease Center, School of Basic Medical Sciences, Beijing University, Beijing, 100083 China; 20 Institute of Hepatology, People’s Hospital, Beijing University, Beijing, 100044 China; 21 Department of Gastroenterology, Zhongshan Hospital, School of Medicine, Fudan University, Shanghai, 200032 China; 22 Department of Pathology, China-Japan Friendship Hospital, Capital Medical University, Beijing, 100029 China; 23 Center of Liver Diseases, Beijing Ditan Hospital, Capital Medical University, Beijing, 100011 China; 24 Department of Gastroenterology, Tongji Hospital, School of Medicine, Tongji University, Shanghai, 200065c China; 25 Department of Infectious Disease, Union Hospital, Tongji Medical College, Huazhong University of Science and Technology, Wuhan, 430022 China; 26 Department of Infectious Disease, Beijing University First Hospital, Beijing, 100034 China; 27 Dongfang Hospital, Beijing University of Chinese Medicine, Beijing, 100078c China

**Keywords:** Drug-induced liver injury, Epidemiology, Pathogenesis, Pathology, Clinical type, Diagnosis, Differential diagnosis, Treatment, Prevention, Recommendations

## Abstract

Drug-induced liver injury (DILI) is an important clinical problem, which has received more attention in recent decades. It can be induced by small chemical molecules, biological agents, traditional Chinese medicines (TCM), natural medicines (NM), health products (HP), and dietary supplements (DS). Idiosyncratic DILI is far more common than intrinsic DILI clinically and can be classified into hepatocellular injury, cholestatic injury, hepatocellular-cholestatic mixed injury, and vascular injury based on the types of injured target cells. The CSH guidelines summarized the epidemiology, pathogenesis, pathology, and clinical manifestation and gives 16 evidence-based recommendations on diagnosis, differential diagnosis, treatment, and prevention of DILI.

## Background

Drug-induced liver injury (DILI) refers to liver injury induced by all types of prescription or non-prescription drugs, including small chemical molecules, biological agents, traditional Chinese medicines (TCM), natural medicines (NM), health products (HP), and dietary supplements (DS) [1–4]. TCM refer to Chinese traditional medical herbs and non-herbal substances, their prepared slices, or prepared compounds composed of multiple herbs and/or non-herbal components produced under the guidance of TCM theories. NM refer to natural medicinal substances or their active constituents, which are prepared using modern technologies. HP refer to products containing vitamins, mineral substances, or other chemical components believed to benefit human health. Such products are not for the treatment of specific disease and are not classified as drugs, and they are believed to have no toxicity. DS refer to substances intended to supplement the diet, but not to constitute a complete meal [3]. Complementary and alternative medications (CAMs), a collective term for a variety of widespread used nutritional and natural medical approaches, include multivitamins, herbs, dietary supplements, bodybuilding agents, and weight loss supplements [5–7]. In fact, CAMs have the same meaning of TCM-NM-HP-DS, but the latter may be more intuitive and easily understood.

DILI is one of the most common and serious adverse drug reactions (ADR) [1, 8]. When severe, it may cause acute liver failure (ALF) and even death [9]. So far there is still lack of indexes for easy, objective, and specific diagnosis, as well as specific treatments for DILI.

The United States (US) established the Drug-Induced Liver Injury Network (DILIN) in 2003 and initiated a DILIN prospective study in 2004 [2]. DILIN helped create the LiverTox website (http://www.livertox.nih.gov) [10], which was established in 2012. The American College of Gastroenterology (ACG) issued the first clinical guideline targeting the diagnosis and management of idiosyncratic DILI (IDILI) in 2014 [3]. In the same year, China published the HepaTox website (http://www.hepatox.org) [11]. Information about liver injury caused by nearly 700 and over 400 types of common drugs are separately recorded in LiverTox and HepaTox websites, respectively. Such information provides clinicians with an important basis for prudent prescription of potentially hepatotoxic drugs, as well as the assessment of their respective risks and benefits.

To help clinicians better understand DILI, and to help them execute relevant research work and avoid confusion in the diagnosis and treatment of DILI, the Chinese Society of Hepatology (CSH), a branch of the Chinese Medical Association (CMA), organized appropriate Chinese experts to draft a DILI guidelines with the goal of standardizing the diagnosis and management of DILI in China. This manuscript is the product of this effort. As new data emerges from DILI research, the guidelines will be updated at a proper time in the future.

This Guideline adopts the Grading of Recommendations Assessment, Development and Evaluation (GRADE) system to assess the grading strength of recommendations (shown in Table 1) and the quality of evidence (shown in Table 2).

**Table 1:** Strength of recommendations in the GRADE system

Strength of recommendations	Description
Strong recommendations (Grade 1)	Intervention measures show explicitly that advantages outweigh disadvantages, or just conversely
Weak recommendations (Grade 2)	Advantages and disadvantages are uncertain or equivalent as shown by the evidence with any quality

**Table 2:** Quality of evidence and its definition in the GRADE system

Quality of evidence	Definition
High quality (A)	We are very confident that the true effect lies close to the estimate of the effect. Further research is very unlikely to change our confidence in the estimate of effect
Moderate quality (B)	We are moderately confident in the effect estimate: the true effect is likely to be close to the estimate of the effect, but there is still a possibility that it is substantially different, further research is likely to have an important impact on our confidence in the estimate of effect and may change the estimate
Low quality (C)	Our confidence in the effect estimate is limited: the true effect may be substantially different from the estimate of the effect. Further research is very likely to have an important impact on our confidence and is likely to change the estimate
Very low quality (D)	We have very little confidence in the effect estimate: the true effect is likely to be substantially different from the estimate of effect. Any estimate of effect is very uncertain

In forming our recommendations, we considered not only the quality of evidence, but also the balance among the advantages and disadvantages, burdens of interventions, the variability of patient preferences and values, the rational use of resources, and the fairness and practicability of the recommended measures.

## Epidemiology

### Incidence and epidemiological trend

In developed countries, the incidence of DILI in the general population is estimated to fall between 1/100,000 and 20/100,000 [1, 12]. Data from France and Iceland suggest that the annual incidence of DILI is approximately 13.9/100,000 and 19.1/100,000 respectively [1, 13]. The incidence of DILI currently reported by China mainly stems from inpatients or outpatients in relevant medical centers [9, 14, 15], of whom patients with acute DILI account for approximately 20% of inpatients with acute liver injury [9]. Because of a lack of large-scale epidemiological data on DILI for the general population, an accurate estimate of the incidence of DILI in the general population of China is not available.

In China, the incidence of DILI tends to rise year by year for the following reasons: Firstly, China has a gigantic population base which takes a wide and growing variety of drugs and TCM-NM-HP-DS. Secondly, persons misuse drugs frequently because many TCM-NM, and nearly all the HP-DS, can be obtained without a prescription and used as the patient likes. Thirdly, medical workers and the public still have insufficient understanding of drug safety issues, including DILI [9]. In addition, DILI types and incidence may vary across different geographical regions of China [9, 14, 15] with distinct drug types and medication practices (involving the dose and the course of treatment), performance of ADR reporting system, as well as genetic polymorphism of drug metabolizing enzymes in different ethnic groups and populations [12].

### Drugs causing DILI

Many marketed drugs have potential to cause hepatotoxicity; the common types of drugs causing DILI include nonsteroidal anti-inflammatory drugs (NSAIDs), anti-infective drugs (including antituberculosis drugs), anti-cancer drugs, central nervous system drugs, cardiovascular system drugs, drugs used for metabolic disorders, hormonal drugs, certain biological preparations, as well as TCM-NM-HP-DS [3, 13]. Different drugs can lead to the same type of liver injury, although DILI due to a specific drug often has a characteristic clinical presentation or “signature”. The same drug can also lead to different types of liver injury. For detailed information, please refer to the HepaTox and LiverTox websites.

In the developed countries of Europe and North America, NSAIDs, anti-infective drugs, and herbal and dietary supplements (HDS) are common causes of DILI, among which acetaminophen (APAP) is the predominant cause of ALF [16, 17]. DILI related to TCM-NM-HP-DS or HDS has received more and more attention in the world in recent years. In 2013, a prospective study from Iceland indicated that HDS accounted for 16% of the causes of DILI [1], while recent DILIN (American) data showed that HDS accounted for over 20% of the causes of DILI. It is reported in China that the top causes of DILI were TCM (23%), anti-infective drugs (17.6%), anti-cancer drugs (15%), hormonal drugs (14%), cardiovascular drugs (10%), NSAIDs (8.7%), immunosuppressive agents (4.7%), and sedative and neuropsychiatric drugs (2.6%) [9].

In China, the TCM-NM-HP-DS that are mostly reported to be related to liver injury including tuber fleece flower root, gynura segetum, and certain compound agents for treating diseases such as osteoporosis, arthritis, vitiligo, psoriasis, eczema, and acne. However, the drug components are complex, so it is difficult to determine which ingredient causes liver injury [3]. According to the *Provisions for Drug Registration*, TCM preparations (TCMp) ready for convenient clinical application and usually in the form of capsules, pills, or tablets, are required to undergo pharmaceutical, pharmacological, toxicological, and clinical research and strict assessment before approval for marketing, thus ensuring that such TCMp meet the criteria for clinical safety and effectiveness. The *Chinese Pharmacopoeia* stipulates that all slice-type Chinese traditional drugs, except the ones used as both drugs and food, must be administrated as prescription drugs. Both TCMp and slice-type drugs must be produced and sold according to the *Good Manufacturing Practic*e (GMP) and *Good Supply Practice* (GSP). In contrast, for paste-type drugs and juice-type drugs (i.e., decoctions), which present as liquid medicine extracted from multiple herbals by mixing and boiling them together, both can be prescribed by qualified clinicians without the need for any approval, though they are classified as prescription drugs. In addition, many non-prescription TCM-NM and folk-proven therapeutic recipes are widely used. Meanwhile, HP-DS can be purchased more easily. In the US, a vast majority of HDS are not researched and developed according to the drug standards. There is no mandatory need for them to undergo preclinical and clinical safety and efficacy testing. They can also be marketed without the approval of the Food and Drug Administration (FDA) [10]. All aforementioned factors increase the risks for DILI caused by TCM-NM-HP-DS or HDS. Therefore, the European Union (EU) has already required that all HDS should be registered in strict compliance with *EU Directive on Traditional Herbal Medicinal Products* before marketing.

### Risk factors

#### Host factors

Host factors include genetic factors and non-genetic factors.

A genetic factor refers to a correlation between DILI risk and a genetic polymorphism or variant involving drug metabolizing enzymes, drug transport proteins, and the human leukocyte antigen (HLA) system [3]. Patients of different races may have varied genetic susceptibility to DILI [18].

Although there are multiple non-genetic risk factors (as follows), none have been found to be an important risk factor for liver injury induced by all suspicious drugs.

1. Age: Advanced age may be an important predisposing factor for DILI [19]. However, data from Iceland have suggested that relatively higher DILI incidence in the elder population may be explained by the increased number of drugs taken [20].
2. Sex: Females may show higher susceptibility to certain drugs such as minocycline and methyldopa, and they are prone to show the characteristics of autoimmune hepatitis (AIH) [21]. Also, liver injury caused by TCM-NM-HP-DS [22] is seen more frequently in females.
3. Pregnancy: The commonly suspected drugs that cause DILI during pregnancy include methyldopa, hydralazine, and propylthiouracil (PTU). PTU can cause fulminant hepatitis in pregnant women, which has a high mortality rate [23].
4. Underlying diseases: There is limited evidence that patients with chronic liver disease are more prone to have DILI. However, once it occurs, there is a higher risk for the appearance of liver failure or even death [24]. It is suggested that hepatitis B virus (HBV) or hepatitis C virus (HCV) infection can increase the risk of DILI caused by ARV or antituberculosis drugs. Human immunodeficiency virus (HIV) infection is a predisposing factor for certain types of DILI, and it is also an important factor that influences DILI incidence and mortality in the HIV-infected patients [25, 26].

It is still unknown whether autoimmune liver disease, non-alcoholic fatty liver disease (NAFLD), or obesity can increase the risk for DILI [27], but patients with autoimmune-like DILI might have higher risk to develop chronic DILI. Diabetes is a predisposing factor for DILI caused by certain drugs and is independently associated with the severity of DILI. Tumors and heart disease are also possible risk factors for chronic DILI [18]. It was reported that patients treated with central nervous system and cardiovascular drugs were more frequent among the group with chronic DILI than the group with self-limiting DILI, and the difference may be attributed to the persistent use of corresponding culprit drugs [28].

#### Pharmaceutical factors

The drug’s chemical properties, dosage, and treatment course, as well as interactions among drugs can often affect the latent period, clinical phenotype, duration, and outcome of DILI. A type of drug can change the absorption, distribution, metabolism, excretion, and pharmacological action of other drugs. The interaction among drugs is a factor for greater risk of DILI, which cannot be neglected, e.g., DILI incidence will increase when some antituberculosis drugs are used simultaneously with some other drugs including azole antifungal drugs, methotrexate, antispasmodic drugs, halothane, or APAP [29, 30]. Also, the contamination of traditional Chinese medicinal materials during preparation may be an important factor for greater risk of occurrence of DILI.

#### Environmental factors

Excessive alcohol consumption may increase the risk of DILI caused by duloxetine, APAP, methotrexate, and isoniazid [3]. The impact of smoking on the susceptibility to DILI is still unknown.

## Tolerators, adaptors, and non-adaptors to a hepatotoxic drug

Different people may react differently when they are exposed to a certain potential hepatotoxic drug. No detected liver injury will be found in the tolerators or nonsusceptibles. Mild and transient liver injury, which can recover naturally even when the culprit drug is continued, may be detected occasionally in adaptors. Adaption to a hepatotoxic drug has been found in many cases, such as for users of isoniazid [31], tacrine, and many other drugs [32–34]. Clinically significant liver injury, which may be reversible or irreversible after drug withdrawal, will be present in non-adaptors.

### Pathogenesis

DILI has a complex pathogenesis and tends to result from a sequence of effects or joint simultaneous effects through several mechanisms, which, so far, are not yet fully elucidated. Usually, the pathogenesis of DILI can be generalized as a mechanism of direct hepatotoxic effects or idiosyncratic hepatotoxic effects. Both processes involve “upstream” events caused by drugs, as well as their metabolic products and “downstream” events caused by the imbalance between pathways to injury and protection of target liver cells.

The direct hepatotoxicity of drugs refers to the direct injury to the liver caused by the ingested drugs and/or their metabolic products. Also known as intrinsic DILI (InDILI), it often appears to be dose-dependent and is usually predictable in animal models [35, 36]. The direct hepatotoxicity of drugs can further cause other mechanisms of liver injury that involve immune and inflammatory responses.

The mechanism of idiosyncratic hepatotoxic effects is a hot research topic in recent years. Genetic polymorphisms can contribute to dysfunction in relevant enzymes and transport proteins such as drug-metabolizing enzymes (including phase I metabolic enzymes such as cytochrome P450 and various phase II metabolic enzymes), transmembrane transport proteins (including ATP-binding cassette protein B11), and solute transport proteins (including organic anion transporting polypeptide 1B1) [37–40]. In addition, the HLA polymorphism may cause the human body to be prone to producing adaptive immune responses to the liver in response to drugs [41].These genetic polymorphisms and their phenotypic and genetic features can increase the host's susceptibility to DILI. Drugs and corresponding reactive metabolites can lead to damage of hepatocellular mitochondria and induce oxidative stress, thus causing hepatocellular injury or death via various molecular mechanisms [42–49]. The endoplasmic reticulum stress response (ERSR) can also promote the progression of DILI [50–54]. Drugs and their metabolites can activate multiple types of death signaling pathways, thus promoting apoptosis, necrosis, and autophagic death [55–58]. An adaptive immune attack may be the final common event of IDILI. First, danger signals produced by cell injury and death can activate antigen-presenting cells and then induce an adaptive immune attack. Second, many metabolites of drugs may act as haptens and bind to host proteins to form neoantigens. If adaptive immune responses target host proteins in neoantigens, they will contribute to autoimmune responses, and if they recognize the metabolites of drugs in neoantigens, they will contribute to anti-drug immune responses [59–65]. In addition, adaptive immune responses can mediate IDILI and also lead to extrahepatic immune injury and then produce systemic manifestations including fever and rashes. Inflammatory responses are mainly a combination of immune activation and a series of related cellular and molecular events. The interaction between inflammation and drug exposure is an important hypothesis about DILI pathogenesis. Intrahepatic inflammation caused by non-drug factors is an independent predisposing factor for DILI and also a factor that promotes the progression of DILI [66]; on the other hand, drugs and their metabolites can also trigger intrahepatic inflammatory responses and promote the progression of DILI [66–70]. Finally, it should be noted that when initiating liver injury, drugs will also trigger restorative tissue repair (RTR) [71, 72]. After the initiation of liver injury, if there is a lack of RTR, injury will rapidly develop; on the contrary, if there is timely and adequate RTR, liver injury will be limited and reversed. Therefore, RTR is an important determining factor for the progression or resolution of liver injury [72].

### Pathology of DILI

In DILI, the injured target cells are mainly hepatocytes, bile duct epithelial cells, and vascular endothelial cells of the hepatic sinusoids and intrahepatic venous system, and these cells can be injured in various complex ways. The histological changes in DILI may mimic almost all of the changes observed in other liver diseases. In some patients with DILI, the implicated drugs and patterns of liver injury are relatively fixed, while in most patients with DILI, there are only individual case reports on liver injury caused by certain drugs and limited information on liver biopsy. Histological changes should be assessed in combination with the patient’s clinical manifestations and history of drug administration. The patterns and severity of liver injury should also be described, which is of crucial importance for definite diagnosis and prognosis.

The diverse histopathological patterns of DILI and some instructional recommendations for the assessment and description of DILI severity have been summarized by Kleiner somewhere else [73]. The patterns of injury are useful for the differential diagnoses, because when known, most drugs have certain correlations with limited patterns of liver injury [74–76]. The patterns of injury can also suggest pathophysiological mechanisms; for instance, diffuse microvesicular steatosis of the hepatocytes suggests mitochondrial injury [77], and zonal necrosis of the hepatocytes suggests the presence of toxic metabolites or vascular injury [78]. Because of the diversity of pathological manifestations of DILI, there is no uniform severity classification system available currently.

## Clinical types and manifestations of DILI

### Clinical types

#### InDILI and IDILI

Based on the pathogenesis, DILI is classified into InDILI and IDILI. InDILI is usually predictable, closely correlated with the drug dose, with a short latency period. The dose required to cause liver injury may vary among patients, but virtually all patients will develop the liver injury at a sufficient dose. InDILI is very rare now, and only drugs with benefits obviously outweighing risks can be approved for marketing. Unlike InDILI, IDILI is unpredictable, clinically common, has diverse clinical manifestations, and generally will not cause liver injury in most individuals even at high doses. IDILI liability is generally not detected in animal experiments [35]. Multiple types of drugs can cause IDILI [3, 79].

IDILI can be further classified into immune-mediated IDILI and genetically meditated IDILI. Immune-mediated IDILI has two types of manifestations, i.e., hypersensibility and drug-induced autoimmune injury. The former usually occurs rapidly (at 1–6 weeks after drug administration), clinically manifests as fever, rashes, increased eosinophils, and can rapidly lead to liver injury if the drugs are re-administered. The latter occurs slowly, and usually without fever, rashes or increased eosinophils. Autoantibodies characteristic of autoimmune liver diseases including AIH or primary biliary cholangitis (PBC) and primary sclerosing cholangitis (PSC) may be present. Genetically meditated IDILI usually has no characteristics of the immune response. It typically does not occur until weeks or months on treatment (>1 year is unusual) and may not rapidly lead to liver injury when the drugs are re-administered [3, 79].

#### Acute DILI and chronic DILI

Based on the course of disease, DILI is classified into acute DILI and chronic DILI. This guideline adopts the following definition of chronic DILI: within 6 months after DILI occurs, serum ALT, AST, ALP, or TBil still remain abnormal, or there is radiographic and histological evidence for portal hypertension or chronic liver injury [2, 3, 80]. Clinically, acute DILI accounts for the vast majority DILI patients, of whom 6–20% can develop into chronic DILI [6, 18, 81, 82]. It is shown that within 3 months after the onset of acute DILI, approximately 42% of patients still had abnormal hepatic biochemical tests, and 1 year later, approximately 17% of patients still had abnormal hepatic biochemical indexes [83]. Cholestatic DILI is relatively prone to develop into chronic DILI [27].

#### Hepatocellular DILI, cholestatic DILI, mixed DILI, and drug-induced vascular liver injury

Based on the type of injured target cells, DILI is classified into hepatocellular injury, cholestatic injury, hepatocellular-cholestatic mixed injury, and vascular liver injury.

The criteria for judging the former three types of DILI, preliminarily established and then revised by the Council for International Organizations of Medical Sciences (CIOMS), are [3, 79, 84]: (1) hepatocellular injury, ALT ≥3 ULN and *R* ≥ 5; (2) cholestatic injury, ALP ≥2 ULN and *R* ≤ 2; (3) hepatocellular-cholestatic mixed injury, ALT ≥3 ULN, ALP ≥2 ULN and 2 < *R* < 5. If ALT and ALP do not reach the aforementioned criteria, the patient’s condition is called “liver biochemical test abnormalities”. *R* = (actual ALT/ALT ULN)/(actual ALP/ALP ULN). Calculating the *R* value at different times during the course of the treatment helps to judge more accurately the clinical types and evolution of DILI. Recently, a “new *R* value (NR value)” is proposed, which is different from *R*, in that the higher value of ALT or AST is taken for calculation [85]. Cholestatic DILI accounts for approximately 30% of total DILI cases, but this percentage probably underestimates the true incidence [71].

Vascular liver injury is relatively rare with unknown pathogenesis, in which the target cells can be endothelial cells of the hepatic sinus, hepatic venules, as well as the hepatic vein and the portal vein, and the clinical types include sinusoidal obstruction syndrome (SOS)/hepatic veno-occlusive disease (VOD) [86, 87], peliosis hepatis (PH) [88, 89], Budd–Chiari syndrome (BCS), idiopathic portal hypertension (IPH) induced by portal sclerosis, and venous, as well as nodular regenerative hyperplasia (NRH) of the liver [73]. Drugs causing vascular liver injury include herbal medicines containing pyrrolizidine alkaloids, certain chemotherapy drugs, anabolic hormones, contraceptives, immunosuppressive agents, and ARV drugs, the targeted vascular endothelial cells of which are different or overlapping. For instance, SOS/VOD is associated with the injury to the endothelia of the hepatic sinus and terminal hepatic venules. Clinically, it is caused mainly by large-dose radiochemotherapy [87] and herbs containing pyrrolizidine alkaloids such as gynura segetum [86]. In the past decade, China has reported over 100 cases of SOS/VOD caused by gynura segetum and other alkaloids. It should be noted that vascular injury can also be caused by infections and immune disorders.

#### DILI-related benign and malignant liver tumors

It has been suggested that certain drugs may be associated with various benign and malignant liver tumors, e.g., focal nodular hyperplasia (possibly related to long-term oral contraceptive use), hepatocellular adenoma (possibly related to long-term use of sex steroids such as androgens, contraceptive steroids, or danazo), hepatocellular carcinoma (possibly related to sex steroids, arsenicals, or thorotrast), cholangiocarcinoma (possibly related to sex steroids or thorotrast), and angiosarcoma (possibly related to sex steroids, arsenicals, thorotrast, or vinyl chloride). Currently, these correlations are mainly of epidemiologic interest [73].

### Clinical manifestation of DILI

The clinical manifestations of acute DILI are usually non-specific. The latent periods of acute DILI vary greatly across individuals, which may be as short as 1 to several days or as long as several months. Most patients with acute DILI may have no significant symptoms and only present with varying elevations in the level of hepatic biochemical indexes including serum ALT, AST, ALP, and GGT. Some patients with acute DILI may have symptoms such as fatigue, decreased appetite, aversion to oily food, tender liver, and epigastric discomfort [3, 9]. Those with significant cholestasis may have jaundice, light-colored faeces, and pruritus. A few patients may have allergic manifestations including fever, rashes, increased eosinophils, and even aching pain in joints, which may be accompanied by other manifestations of extrahepatic organ damage. Some patients may develop into ALF or subacute liver failure (SALF).

Clinically, chronic DILI may manifest as chronic hepatitis, liver fibrosis, compensated and decompensated cirrhosis, AIH-like DILI, chronic intrahepatic cholestasis, vanishing bile duct syndrome (VBDS), and so on. A few patients may also present with SOS/VOD or liver tumors. SOS/VOD may appear acutely with ascites, jaundice, and hepatomegaly [79].

## Laboratory, imaging, and pathologic examination

### Laboratory tests

Most DILI patients have no significant changes in the routine blood tests compared with the baseline values. Patients with allergic and idiosyncratic reactions may possibly present with elevated percentages of eosinophils (>5%). Attention should be paid to the impacts of underlying diseases on patients’ hematological indexes.

Currently, the changes of serum ALT, ALP, GGT, and TBil are the main laboratory indexes for judging whether there is liver injury and the severity of DILI. The assessing elevations in serum ALT may be preferable over AST in the diagnosis of DILI [90], because it has higher sensitivity and specificity than AST in detecting liver injury. Serum ALT level may be over 100 ULN in some patients with DILI. However, it should be noted that some patients with DILI may not present with significantly elevated ALT levels. For instance, although high elevations in serum ALT were found in some patients treated with tacrine, many other patients who took tacrine only showed slight elevations of ALT levels and did not develop more severe liver injury. On the other hand, rare forms of liver injury, such as Reye’s syndrome-like effects caused by aspirin can cause severe liver injury without large elevations in serum ALT or AST.

With regard to serum ALP elevation, non-liver sources of ALP elevation should be ruled out both in children in the period of growth and development and patients with bone diseases. The sensitivity and specificity of serum GGT may be useful in distinguishing liver origin of ALP as it is relatively liver specific and rises in cholestatic injuries.

Elevated serum TBil, dropped albumin levels and blood coagulation dysfunction suggest severe liver injury. However, kidney disease, systemic inflammation, and malnutrition, which can result in the dropped albumin levels, and hematological disease, which can result in blood coagulation dysfunction, should be ruled out. Usually, the increase of international normalized ratio (INR) and decrease of prothrombin activity (PTA) serve as rational indexes of blood coagulation dysfunction.

### Imaging examination

In patients with acute DILI, the liver usually has no significant changes on imaging or only mild hepatomegaly. Patients with drug-induced ALF may have decreased liver volumes as the disease progresses. Patients with chronic DILI usually do not have significantly dilated intra- and extra-hepatic bile ducts, but some may have changes consistent with cirrhosis, including splenomegaly and enlarged internal diameter of portal vein. Imaging examinations are often helpful to diagnose SOS/VOD, a plain computed tomography (CT) scan may show a swollen liver, and an enhanced CT scan during portal venous phase may show uneven or patchy change of the liver images, blurred hepatic veins, and ascites [86]. Routine imaging examinations including ultrasound, CT, or MRI scan, as well as retrograde cholangiopancreatography are of great value for distinguishing cholestatic DILI from biliary obstruction cause such as caused by gall stones biliary or pancreatic malignancies.

### Novel biomarkers for DILI

A desirable biomarker for DILI should be helpful for detecting subclinical DILI, improving the clinical diagnosis of DILI, identifying the severity of DILI, distinguishing adaptive DILI from progressive DILI to predict the prognosis of DILI. However, in current clinical practice, none of the commonly used indexes such as ALT, ALP, TBil, and INR show specificities for the diagnosis of DILI, though they can help judge the severity of DILI and in some cases, its prognosis [3, 85, 91].

In recent years, several new types of DILI-related serological, biochemical, and histological biomarkers have been reported, including apoptosis-related cytokeratin-18 fragments (CK-18Fr) [92], soluble Fas and Fas ligand (sFas/sFasL), soluble TNF-α and TNF receptor (sTNF-α/sTNFR), and soluble TNF-related apoptosis-inducing ligand (sTRAIL) [92]; cell necrosis-related full-length CK-18 (CK-18FL), high-mobility group protein B1 (HMGB1) [92], and MicroRNA (especially miR-122) [93–98]; specific mitochondrial biomarkers [92, 98]; circulating autoantibodies targeting drug-metabolizing enzymes such as CYPs [85, 97]; biomarkers reflecting cholestasis [99], as well as genetic biomarkers reflecting the susceptibility to DILI, such as the genetic polymorphisms of HLA, drug-metabolizing enzymes and drug transport proteins [93, 94]. However, the aforementioned markers have poor specificities for the diagnosis of DILI, and their value for clinical use still needs to be widely verified. Currently, it is only found that APAP-protein adducts [3, 97] are specific biomarkers of APAP-mediated DILI [94, 100], and pyrrole-protein adducts appear to be important biomarkers of SOS/VOD caused by gynura segetum [86, 101].

### Histopathological examination

When a series of clinical investigations and laboratory tests still cannot yield a confident diagnosis of DILI, a liver biopsy may be useful for diagnosis and assessment of the severity of liver injury.

## Diagnosis and differential diagnosis

Currently, the diagnosis of DILI is one of exclusion. Firstly, the presence of liver injury should be confirmed. Secondly, liver injury caused by other insults should be excluded. Finally, causality assessment should be performed by relating the injury and its characteristics to the specific drugs that patient had taken.

### Key points for the diagnosis of DILI

It is very important to trace comprehensively and cautiously the history of the suspected drug use and exclude other causes of liver injury to establish the diagnosis of DILI, because the onset time of DILI varies greatly, and there is a lack of specific biomarkers for DILI.

DILI occurring in a patient with preexisting liver disease is prone to be misread as flare or aggravation of the preexisting liver disease. It was reported that over 6% of DILI patients had a past history of liver disease [82], and approximately 1% of such patients might present with DILI [102]. For instance, when patients with HBV or HCV infection complicated by inflammatory bowel disease (IBD) receive immunosuppressant therapy, they are prone to develop into liver injury [103], but it is difficult to identify whether the liver injury is caused by hepatitis virus activation, or autoimmune liver injury complicating IBD, or it is DILI caused by immunosuppressant drugs, or even the three conditions occurring simultaneously. Thus, when there are several possible coexisted causes of liver injury, it is very important, but usually difficult to identify the exact cause of liver injury. It is believed that both the incidence and severity of DILI occurring in patients with underlying liver disease may have been underestimated.

Drug-related self-limiting liver injury occurs in many patients (i.e., adaptors) [104], and complete recovery is expected, so it may not be necessary to discontinue the drug if it is important for the controlling of primary disease and equally effective alternative therapies are not available. To avoid unnecessary drug withdrawal, the International Serious Adverse Events Consortium (iSAEC) recommended in 2011 the modified biochemical criteria for the identification of DILI as reaching any of the following items [105]: (1) ALT ≥5 ULN; (2) ALP ≥2 ULN, especially in patients with elevated 5′-nucleotidase or GGT, and without bone-diseases-related ALP elevation; (3) ALT ≥3 ULN and TBil ≥2 ULN.

Liver biopsy should be considered if any of the following items is met: (1) the diagnosis of DILI remains uncertain after the appropriate laboratory tests have not identified the etiology, and especially when AIH cannot be ruled out; (2) though suspected hepatotoxic drugs are stopped, the levels of hepatic biochemical indexes continue worsening or other signs of aggravated liver function appear; (3) the levels of hepatic biochemical indexes do not drop to ≤50% of the peak values after cessation of suspected drug for 1–3 months; (4) suspected existence of chronic DILI or other accompanying chronic liver disease; (5) long use of certain drugs which have the risk to cause liver fibrosis, e.g., methotrexate.

### Causality assessment methods

The most commonly used tool for the diagnosis of DILI is the Roussel Uclaf causality assessment method (RUCAM) [106–108], of which the original form can be dated back to 1989 and called as the Council for International Organizations of Medical Sciences (CIOMS) scale. It was well established as a liver-specific quantitatively causality assessment method in 1993 [106], and has been updated recently by Danan et al. [108]. Though subsequently there were several other assessment methods [109], the practice has proved that the RUCAM remains the most rational, comprehensive, and convenient tool with a relatively higher rate of accurate diagnosis for DILI [3]. RUCAM has the following advantages: (1) It is not affected by age, sex, or race; (2) the selected parameters are comprehensive, relatively rational, and objective, and the semi-quantitative analysis system based on specific questions can be understood and applied even by non-hepatology clinicians; (3) The method differentiates the scoring standards for different types of DILI. The disadvantages of RUCAM include: some scoring standards are vaguely defined, the parameters and their weight need to be improved, and the instructions for completing each item in the RUCAM table should be more detailed and clear [106–108]. In addition, the characteristic clinical presentations, or signatures, for DILI due to specific drugs are not considered.

Experience from other reported causality assessment tools is limited, such as Maria & Victorino method [109, 110], Naranjo scoring system [111], drug lymphocyte stimulation test (DLST) as a supplement to RUCAM from Japan [112], and a previous simplified method from China [113]. In the US, DILIN proposed a structured expert opinion procedure (SEOP) [114], but there are discrepancies between the outcomes of SEOP and RUCAM. The SEOP may be superior in certain situations; however, it has a complicated process and requires three expert hepatologists, making it impractical for daily clinical practice.

The guidelines recommend the use of RUCAM [106–108] to make a comprehensive assessment of the causality between drugs and liver injury, and the following data need to be collected in detail: (1) the history of drug administration, especially the interval from drug initiation to the onset of liver injury; (2) the duration of illness and the dynamic features of biochemical abnormalities; (3) patient risk factors; (4) other concomitant drug treatments; (5) exclusion or weight of nonpharmaceutical factors related to liver injury, and exclusion of nonhepatic factors that can alter liver chemistries. For other causes of liver injury to be excluded, except AIH, PBC, PSC, chronic hepatitis B (CHB), and chronic hepatitis C (CHC), which have been listed in the RUCAM Scale Table, diseases with lower incidence such as acute hepatitis E and IgG4-related cholangitis should also be excluded in China; (6) previous information on hepatotoxicity of suspected drugs; (7) reactions on unintended rechallenge of suspected drugs. It is especially emphasized that the intended rechallenge must be prohibited to avoid high risk of severe liver injury. For difficult cases, liver biopsies may be considered.

For RUCAM, the causality between suspected drugs and liver injury is classified into five grades according to the scoring results [106–108]: (1) highly probable ≥9 points; (2) probable 6–8 points; (3) possible 3–5 points; (4) unlikely 1–2 points; (5) excluded ≤0 points.

For SEOP, the causality between suspected drugs and liver injury is classified into six grades according to the assessment results [114]: (1) definite: quantified possibility >95%, any reasonable doubt on the diagnosis can be excluded; (2) highly likely: quantified possibility 75–95%, the evidence of DILI is clear or convincing but not sure; (3) probable: quantified possibility 50–74%, the evidence tends to support the presence of a causality; (4) possible: quantified possibility 25–49%, the evidence is not sufficient to reach a convincing causality, but the diagnosis of DILI cannot be completely excluded; (5) unlikely: quantified possibility <25%, current evidence suggests that the causality is impossible; (6) insufficient information: a valid scoring cannot be performed due to a lack of key evidence.

### Grades of DILI severity

To date, the severity of acute DILI is traditionally classified into five Grades [93], and it has been further revised in the DILIN prospective study [2, 83]. But the thresholds of INR and TBil for the grading of DILI severity are not definite, especially for the judgement of ALF. Therefore, in these CSH guidelines, the grades of DILI severity are further modified to keep accordance with the definitions of ALF [115, 116].

- *Grade 0* (*no liver injury*): Patients tolerate drug treatment and have no hepatotoxic reactions.
- *Grade 1* (*mild liver injury*): Elevations in serum ALT and/or ALP levels, TBil <2.5 ULN (2.5 mg/dL or 42.75 μmol/L), INR <1.5. Most patients show adaptability to the liver injury. Patients may present with or without symptoms such as fatigue, asthenia, nausea, anorexia, right upper abdominal pain, jaundice, pruritus, rashes, or weight loss [3, 117].
- *Grade 2* (*moderate liver injury*): Elevated serum ALT and/or ALP, with TBil ≥2.5 ULN or INR ≥1.5. The above mentioned symptoms may become aggravated.
- *Grade 3* (*severe liver injury*): Elevated serum ALT and/or ALP, TBil ≥5 ULN (5 mg/dL or 85.5 μmol/L) with or without INR ≥1.5. The symptoms are further aggravated, which indicates the need of hospitalization or delayed hospital stay, but there is no evidence of hepatic encephalopathy.
- *Grade 4* (*ALF*): Evidence of coagulation abnormality indicated by INR ≥1.5 [115, 116] or PTA <40% [115], signs of hepatic encephalopathy, and TBil ≥10 ULN (10 mg/dL or 171 μmol/L) or daily elevation ≥1.0 mg/dL (17.1 μmol/L) [115] in 26 weeks after the DILI onset. Patients may have ascites and DILI-related dysfunction of other organs. If there is evidence of underlying chronic liver diseases, especially liver cirrhosis, the diagnosis of acute-on-chronic liver failure (ACLF) is established.
- *Grade 5* (*lethal*): Death due to DILI, or need to receive liver transplantation for survival.

### Diagnostic algorithm

**Fig. 1:**
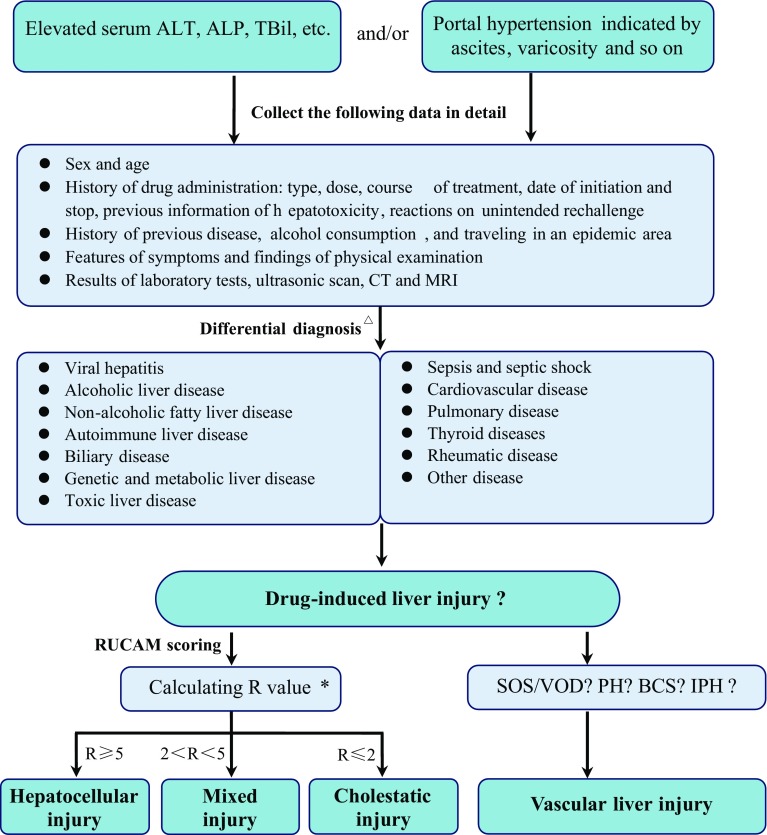
Algorithm for the diagnosis of DILI. *BCS* Budd–Chiari syndrome, *IPH* idiopathic portal hypertension, *NRH* nodular regenerative hyperplasia, *PH* peliosis hepatis, *SOS/VOD* sinusoidal obstruction syndrome/veno-occlusive disease. *Asterisk R* = (actual ALT/ULN)/(actual ALP/ULN).* Triangle* see Table 3

The diagnostic algorithm for DILI is shown in Fig. 1.

### Standard format for the diagnosis of DILI

A complete diagnosis of DILI should include the identified causative drug, the clinical type, whether the course is acute or chronic, RUCAM scoring result, and the grade of severity. Examples of diagnosis are as follows:

- Drug-induced liver injury due to isoniazid, hepatocellular type, acute, RUCAM score 9 points (very likely), Severity Grade 3.
- Drug-induced liver injury due to augmentin, cholestatic type, chronic, RUCAM score 7 points (very likely), Severity Grade 2.

### Differential diagnosis

#### Key points of differential diagnosis

DILI has complex clinical phenotypes, which cover almost all known phenotypes of acute, subacute, and chronic liver injury. The exclusion of other liver diseases (see Table 3) is essential for the diagnosis of DILI. Therefore, any other hepatobiliary diseases, including all types of viral hepatitis (especially sporadic hepatitis E), NAFLD, alcoholic liver disease, AIH, PBC, PSC, Wilson’s disease, α1-antitrypsin deficiency, and hemochromatosis, should be excluded by detailed inquiry of the patient’s history, symptoms, signs, course of illness, etiological detection, biochemical abnormalities, imaging analysis, and histopathological examination if available.

**Table 3:** Differential diagnosis of DILI

Differential diagnosis	Key diagnostic parameters or notes
Viral infections	
Hepatitis A virus (HAV)	Serum anti-HAV-IgM
Hepatitis B virus (HBV)	Serum HBV surface antigen (HBsAg), anti-HBc-IgM; HBV DNA (PCR)
Hepatitis C virus (HCV)	Serum anti-HCV, HCV antigen; HCV RNA (RT-PCR)
Hepatitis E virus (HEV)	Serum anti-HEV-IgM, titer increase for anti-HEV-IgG; HEV RNA (RT-PCR)
Cytomegalovirus (CMV)	Serum anti-CMV-IgM, titer increase for anti-CMV-IgG; CMV DNA (PCR)
Epstein Barr virus (EBV)	Serum anti-EBV-IgM, titer increase for anti-EBV-IgG; EBV DNA (PCR)
Herpes simplex virus (HSV)	Serum anti-HSV-IgM, titer increase for anti-HSV-IgG; HSV DNA (PCR)
Varicella zoster virus (VZV)	Serum anti-VZV-IgM, titer increase for anti-VZV-IgG; VZV RNA (RT-PCR)
Other viral infection	Serum specific biomarkers of Adenovirus, Coxsackie-B virus, Echovirus, Measles virus, Rubella virus, etc
Alcoholic liver disease	History of alcohol abuse. Serum AST/ALT >2, GGT > ULN
Nonalcoholic fatty liver disease	Body mass index, insulin resistance, hepatomegaly, echogenicity of the liver
Autoimmune liver disease	
Autoimmune hepatitis	γ-Globulins, ANA, SMA, anti-LSP, anti-ASGPR, anti-LKM
Primary biliary cholangitis	AMA, anti-PDH-E2. Liver histopathology
Primary sclerosing cholangitis	p-ANCA, MRC. Liver histopathology
Autoimmune cholangitis	ANA, SMA. Liver histopathology
Biliary disease	Obstructive jaundice, increase of serum ALP and GGT; cholangiectasis and inflammation secondary to calculus of bile duct or other factors. Hepatobiliary sonography is usually needed. MRCP and ERCP if necessary
Genetic and metabolic liver disease	
Wilson's disease	Genotyping of Wilson disease; declined serum ceruloplasmin; increased serum free copper; Kayser–Fleischer-ring; neurologic-psychiatric abnormality
Hemochromatosis	Genetic testing of *HFE* gene. Elevated transferrin saturation and ferritin levels liver biopsy to assess the hepatic iron concentration and degree of liver injury
Alpha-1 antitrypsin deficiency	Diminished serum levels of α1-antitrypsin, abnormal mobility of the abnormal α1-AT molecule in isoelectric focusing
Toxic liver disease	Screening for household, occupational and other unexpected toxins
Sepsis and septic shock	White blood cell count and sort, bacterial culture of blood or other sample, etc
Cardiovascular disease	Echocardiogram, electrocardiogram, and clinical context to identify any cardiovascular disease leading to hypotension or shock. Doppler flow imaging to detect thrombosis
Pulmonary disease	CT and clinical context to find pulmonary infarction, COPD or other lung disorders
Thyroid disease	Basal TSH, T3, T4, free T3, free T4, thyroid associated autoimmune antibodies
Rheumatic diseases	Musculoskeletal and skin history and clinical features. Rheumatic factor and autoimmune antibodies. Radiographic, CT and MRI evaluation
Other status	Lymphoma and other oncologic disease; Addison’s disease (plasma cortisol); parenteral nutrition; polytrauma; grand mal seizures; strong physical exercise, etc

CT or MRI is usually needed in patients with hepatomegaly, cirrhosis, liver nodules, vascular liver injury, or biliary duct disorders. Also, liver biopsy is recommended when the cause of liver injury is uncertain

For patients who receive chemotherapeutic or immunosuppressive agents and have positive HBV or HCV markers, if they have liver dysfunction or aggravated liver injury, it should be noted whether this is caused by viral reactivation, or by chemotherapeutic/immunosuppressive agents, or simultaneous occurrence of both [118]. For patients with AIDS who are receiving antiretroviral treatment (ART), if they have coexisting positive HBV or HCV markers and liver injury, it should be identified whether the liver injury is caused by ART or hepatitis virus reactivation.

In addition, anoxic injury secondary to sepsis, poisoning, heart failure, hypotension or shock, vascular occlusion, and pulmonary insufficiency should also be excluded. It should be noted that ascites may be the initial clinical manifestation of SOS/VOD.

#### Identification of DILI from AIH

The clinical features of some DILI patients are similar to those of classic AIH patients, and they may have corresponding autoantibodies related to liver injury and may respond to steroid therapy. Thus, it is clinically difficult to distinguish DILI from AIH in those patients. In general, special attention should be paid to AIH-like DILI (AL-DILI), drug-induced AIH (DIAIH), and DILI occurring on the background of AIH.

AL-DILI is the most frequent phenomenon in the above mentioned three contexts, and its liver injury is usually accompanied by significantly elevated serum immunoglobulin and existence of antinuclear antibody (ANA), anti-smooth muscle antibody (ASMA), anti-liver-kidney microsome antibody-1 (anti-LKM-1), and occasionally antimitochondrial antibody (AMA). Patients with AL-DILI usually have chronic course and AIH-like symptoms, but can also progress to ALF/SALF. Patients with AL-DILI usually respond well to glucocorticoid therapy without liver injury recurrence. In contrast, patients with AIH are expected to continue to require immune suppression. Histopathological examination of liver biopsy is another key approach to distinguishing AL-DILI from classic AIH [119, 120]. The histopathological features of classic AIH include plasma cell infiltration, rosette-like changes of hepatocytes, and lymphocyte emperipolesis, while AL-DILI may have the infiltration of neutrophils and eosinophils in the portal area, as well as hepatocellular cholestasis [119, 120].

For patients who have an onset of liver injury for the first time, with a definite history of drug administration and marked autoimmune features, treatment with glucocorticoid can be considered after the withdrawal of suspected drugs if the liver chemistries do not improve. After the patient is recovered from the liver injury, the dosage of glucocorticoid should be reduced gradually until withdrawal. If there are no signs of recurrence in the follow-up stage, the possibility to diagnosis DILI increases. Otherwise, the diagnosis of classic AIH should be considered [121].

Recommendations 1–5:

1. Currently, the clinical diagnosis of DILI remains one of exclusion and should be performed on a comprehensive analysis of a detailed history of drug administration, clinical features, dynamic changes in liver biochemical tests, response on unintended drug rechallenge if applicable, and exclusion of other causes of liver injury. If necessary, a histological examination of liver biopsies may be helpful to diagnosis (1B)

2. RUCAM is recommended to serve as a semi-quantified score system for the evaluation of causality between suspected drugs. ≥9 points indicate that the correlation between the suspected drug(s) and the liver injury is “highly probable”, 6–8 points indicate “probable”, 3–5 points indicate “possible”, 1–2 points mean “unlikely”, and ≤0 point means “excluded” (1B)

3. Complete diagnosis of DILI should include the name of the implicated drug, clinical type, acute or chronic course, RUCAM score, and grade of severity (1B)

4. It is often difficult to distinguish autoimmune hepatitis (AIH) and AIH-like DILI (AL-DILI). One should carefully collect the history of drug administration, analyze autoimmune indexes, observe the dynamical responses to drug withdrawal and steroid treatment (if given) as well as the course after the withdrawal of immunosuppressive agents, and if necessary, perform a histological examination of the liver for further differentiation (2C)

5. For patients with underlying liver disease or multiple causes of liver injury, more frequent monitoring of liver injury should be undertaken when drugs with potential hepatotoxicity are used. It is necessary to distinguish the natural flare of the underlying disease(s) from DILI, which is important for the correct treatment (1B)

## Treatment of DILI

The principles for DILI treatment [122] are: (1) stop the use of suspected culprit drugs immediately if the drugs are not critical for the control of underlying disease, and avoid using the suspected drugs or similar drugs again; (2) weigh the balance between the progression risk of underlying disease after drug withdrawal and the aggravation risk of liver injury caused by continuous administration of the potentially implicated drugs; (3) treat the DILI with appropriate anti-inflammatory and hepatoprotective agents (AIHPAs) according to the clinical patterns of DILI; (4) emergency liver transplantation should be considered for patients with ALF/SALF.

At present, there is no evidence supporting that the joint application of two or more kinds of AIHPAs will improve therapeutic efficacy for DILI. Therefore, currently this guideline does not recommend the combination of two or more types of AIHPAs to treat DILI. Also, to date there is no evidence supporting the value and necessity of prophylactic use of AIHPAs for reducing the risk of DILI in clinical contexts with potentially increased risk of DILI, such as during anti-tuberculosis or anti-tumor therapy [123]. However, in these clinical contexts, especially within the first three months of anti-tuberculosis therapy, it is very important to do enhanced monitoring of liver-related biochemical tests to detect liver injury earlier and give rational intervention.

### Drug withdrawal

Timely withdrawal of the suspected liver-injuring drugs is the most important treatment strategy for DILI. In most cases, the culprit drugs should be withdrawn immediately after they are identified, and approximately 95% of patients may achieve spontaneous improvement; most will recover completely after the drug cessation, but some patients may develop chronic DILI, and a few cases may progress into ALF/SALF. It is reported that the average recovery course was approximately (3.3 ± 3.1) weeks for patients with pattern of hepatocellular injury, and (6.6 ± 4.2) weeks for patients with pattern of cholestatic injury [9].

Because the individual adaptability to drug hepatotoxicity is common in population, it is believed that an elevation of serum ALT or AST, which is <3 ULN without symptoms is not necessarily an indication for immediate drug withdrawal. But notably, the risk of leading to ALF/SALF will increase with continual use of the hepatoxic drugs when TBil and/or INR have elevated markedly.

In 2009, US FDA formulated some principles for drug withdrawal related to DILI in drug clinical trials [124]. The withdrawal of hepatoxic drugs should be considered if any of the following abnormalities occurs: (1) serum ALT or AST >8 ULN; (2) ALT or AST >5 ULN, which lasts for 2 weeks; (3) ALT or AST >3 ULN, and TBil >2 ULN or INR >1.5; (4) ALT or AST >3 ULN, which is accompanied by gradually aggravated fatigue, nausea, vomiting, right upper abdominal pain or tenderness, fever, rashes, and/or eosinophilia (eosinophils >5%). The aforementioned principles were crafted for subjects enrolled in drug clinical trials and still need to be further validated in prospective studies. Therefore, these principles can only act as a reference for routine clinical practice.

For InDILI, in order to balance the risks between occurrence of liver injury and exaggeration of underlying disease, the dosage of hepatoxic drugs should be reduced if the drugs are essential, and there are no alternative agents for treating the underlying disease.

### Pharmacotherapy

*N*-Acetylcysteine (NAC) can reduce various free radicals [125–128], and the earlier it is used, the better its clinical effectiveness will be. For adult patients, NAC should be given at a dosage of 50–150 mg/(kg day) for at least 3 days; the rate of intravenous infusion should be strictly controlled to avoid some severe adverse reactions. Currently in China, NAC is recommended to treat patients with early stage of ALF on the base of integrated therapy, but it is worthy of future investigation for the therapeutic effects of NAC in patients with moderate or severe DILI. In the US, NAC is the only antidote approved in 2004 by the FDA to treat DILI caused by APAP, and aside from APAP-induced DILI, NAC is only recommended for use in patients with ALF related to non-APAP agents. As shown in a prospective controlled study by an American ALF research group, which was conducted in 24 medical centers for 8 years in 173 patients with ALF caused by non-APAP agents, NAC could improve the survival rate of patients who were at the early stage of DILI-related ALF and had not undergone liver transplantation [7, 129–131]. In 2011, the American Association for the Study of Liver Diseases (AASLD) published guidelines on ALF, which recommended the use of NAC for the treatment of ALF caused by drugs and toadstools [116]. In 2014, the ACG published a guideline for the clinical diagnosis and treatment of IDILI, which recommended the use of NAC for the treatment of patients with early ALF [3]. However, a randomized, controlled treatment trial in children with ALF caused by non-APAP agents did not support a therapeutic role for NAC. Therefore, NAC is not recommended to treat children ALF caused by non-APAP agents, particularly pediatric patients of less than 2 years [3].

Currently, there has been no randomized controlled studies on the therapeutic effect of glucocorticoids on DILI, although they are sometimes used as treatment for very severe DILI. Considering the multiple adverse reactions of glucocorticoids, they should be used with caution. Theoretically and empirically, glucocorticoids could be given to patients with marked signs of hypersensitivity or autoimmunity, but without remarkable improvement or even aggravation of biochemical indicators after the withdrawal of hepatoxic drugs.

Based on the findings that magnesium isoglycyrrhizinate could reduce the serum ALT levels in patients with DILI in randomized controlled trials, magnesium isoglycyrrhizinate recently has been approved by Chinese Food and Drug Administration (CFDA) to treat acute DILI, including the patterns of acute hepatocellular injury and mixed liver injury with significantly elevated serum ALT [132].

Empirically, for the treatment of mild to moderate drug-induced hepatocellular injury or mixed liver injury, bicyclol [133, 134], and glycyrrhizic acid [135] may be considered to treat patients with severe liver inflammation, and silymarin [136] may be selected to treat those with mild liver inflammation. It is reported that both ursodeoxycholic acid (UDCA) [137, 138] and *S*-adenosyl methionine (SAMe) [139–141] have therapeutic effects in patients with cholestatic DILI. The definite therapeutic effects of these drugs are still to be confirmed by prospective randomized and controlled studies.

For SOS/VOD, the early use of anticoagulants such as low-molecular-weight heparins may have some therapeutic effect [87]. For DILI in pregnancy, except the withdrawal of hepatoxic drugs, attention should also be paid to the improvement of pregnancy outcome, prevention of premature delivery, and careful monitoring of the fetus to terminate pregnancy at appropriate time.

### Liver transplantation

Liver transplantation should be considered for patients with ALF/SALF who present with hepatic encephalopathy and severe coagulation disorders, as well as decompensated cirrhosis [7].

Recommendations 6–12:

6. The first general principle for treatment of DILI is prompt discontinuation of suspected hepatoxic drugs. For intrinsic DILI, the hepatoxic drug should be ceased immediately, or the dosage should be reduced when discontinuation of treatment is not desirable and the DILI is mild (1A)

7. To avoid the risks of aggravation or recurrence of underlying diseases treated by the suspected hepatoxic drug(s), the withdrawal of hepatoxic drugs is prudent, but may be necessary when any one of the following occurs: (1) serum ALT or AST >8 ULN; (2) ALT or AST >5 ULN, which lasts for 2 weeks; (3) ALT or AST >3 ULN, with TBil >2 ULN or INR >1.5; (4) ALT or AST >3 ULN, which is accompanied by gradually aggravated fatigue, digestive tract symptoms, and/or increased percentage of eosinophils (>5%) (1B)

8. For early drug-induced ALF and SALF in adult patients, treatment with *N*-acetylcysteine (NAC) as early as possible is recommended. According to the severity of DILI, NAC can be given at 50–150 mg/kg/day for at least 3 days (1A). Currently NAC is not recommended for the treatment of drug-induced ALF and SALF in children patients (2B)

9. Treatment of DILI with glucocorticoids should be cautiously considered; that is, the potential benefits and possible risks of glucocorticoids must be fully weighed. Glucocorticoids are rational for the therapy of immune-mediated DILI, and AIH-like DILI (AL-DILI) with autoimmune features and usually results in a good response, with rare recurrence of liver injury after the withdrawal of glucocorticoid (1B)

10. Magnesium isoglycyrrhizinate can be used to treat acute hepatocellular or mixed DILI with significantly elevated ALT (1A)

11. Among the patients with mild to moderate hepatocellular and mixed DILI, those with severe inflammation in the liver can be treated with bicyclol and glycyrrhizic acid (diammonium glycyrrhizinate enteric-coated capsules or compound glycyrrhizin), and those with mild inflammation can be treated with silymarin. Patients with cholestatic DILI can be treated with ursodeoxycholic acid (UDCA) or *S*-adenosyl methionine (SAMe), although conclusive evidence of benefit is lacking (2B)

12. It is not recommended to combine two or more types of anti-inflammatory and hepatoprotective agents to treat the liver injury, or prophylactically use these drugs to reduce the risk and incidence of DILI (2B)

Liver transplantation should be considered for patients with lethal drug-induced ALF/SALF and decompensated cirrhosis (1B)

## Prognosis of DILI

Most of the patients with acute DILI have a favourable prognosis. Generally speaking, the prognosis of chronic DILI is better than that of chronic liver injury with similar histological changes caused by non-pharmaceutical factors. Cholestatic DILI usually will generally resolve within 3–12 months after the withdrawal of hepatoxic drugs [80], but a few of those patients may have prolonged course and finally develop into severe vanishing bile duct syndrome and cholestatic cirrhosis, which indicates a poor prognosis. A retrospective study of South Korea [142] suggested that the percentage of 30-day poor prognosis after admission was up to 13.1% among 213 patients with DILI. In addition, the Model for End-Stage Liver Disease (MELD) score and the level of hemoglobin were found to be independent predictors for short-term prognosis of those patients, while the clinical patterns of liver injury (hepatocellular injury, mixed or cholestatic pattern) upon admission were less correlated with the short-term prognosis at 30 days.

Drug-induced ALF/SALF has a high mortality rate. The preliminary findings of a US-DILIN multicenter, prospective, and large cohort study showed that among 660 adult patients with drug-related liver injury, 30 cases were given liver transplantation and 32 cases died within 6 months after onset; importantly, the cause leading to death in approximately 53% of the died patients was directly correlated with severe liver injury. Among 133 patients with drug-induced ALF who were enrolled by the US ALF study group, the survival rates of those who did not receive and those who had received liver transplantation within 3 weeks were 23 and 42%, respectively [7].

Dr. Hyman Zimmerman noted that roughly 10% of patients who present with jaundice from hepatocellular DILI, will develop liver failure, and this appears to be generally true regardless of the implicated drug [3, 84]. The US-FDA has taken this observation and defined a “Hy’s Law case”. This is a patient in a clinical trial who develops an elevation in serum ALT or AST >3 ULN together with an elevation in serum TBil >2 ULN. A Hy’s Law Case is viewed by the US-FDA as the “gold standard” liver safety signal. If a single Hy’s Law case occurs, the drug’s hepatotoxicity is worrying; if two such cases appear, this strongly suggests that the drug may cause severe DILI when it is widely used in a population [143]. In a clinical trial of dilevalol, two cases of 1000 subjects met with the criteria of Hy’s Law, and this led to the disapproval of dilevalol by US-FDA. Later, dilevalol was marketed in Portugal and found to be able to cause fatal liver injury. Because of the occurrence of one Hy’s Law case in a clinical trial of tasosartan, the sponsor was required to provide more data on the safety of tasosartan before its entering into market, and this subsequently led to the abandonment of tasosartan.

Recommendation 13:

13. Hy’s Law is of great value for assessing the liver safety of new drugs. If there are Hy’s Law cases in the drug clinical trial data, great attention should be paid to the hepatotoxicity of that drug (1B)

## Prophylaxis and administration, as well as future of DILI

### Prophylaxis and administration

China has a huge population, and the irregular use of drugs is relatively common in clinical practice. Chinese patients generally believe that TCM-NM-HP-DS and other products from natural plants are harmless to health. There is also a lack of sufficient understanding and alertness regarding DILI by medical service providers and the public. All these factors together lead to the current serious status of DILI prevention, which needs systematic strategies to reduce the overall risks of DILI. Currently, multiple ways have been used for the control of risks of DILI as follows:

1. Black-box warnings, precautions, and prevention measures against the hepatotoxicity of drugs in their instructions.
2. Close monitoring of ADRs when the drugs enter into market, and applying intensively the pharmacovigilance concept in the process of drug monitoring and assessment [144]. Currently, China has established a nation-wide ADR monitoring system that includes 34 provincial centers of ADR monitoring, 200,000 grassroots users, and over 6,600,000 individual case reports. ADR case reports can be submitted to the monitoring centers by grassroots users voluntarily, and this regulation provides a favourable guarantee of technology and system for timely discovery of and rapid response against ADRs [145].
3. Prescribing drugs in accordance with related clinical guidelines [146–148]. Controlling the dosage and course of prescribed drugs, and avoiding drug abuse.
4. Monitoring the changes of liver biochemical tests periodically during treatment with drugs known to have liver safety liabilities.
5. Enhancing the management of informed consent for medication and urging patients to keep alert to the risks of DILI.
6. Improving the public health education on safe drug administration, especially attempting to eliminate the wrong concept that the TCM-NM-HP-DS are always safe.

If reliable novel biomarkers for prediction of susceptibility and aggravation of DILI can be found, verified, and put into clinical use in the future, they will help a lot in the control of DILI risk.

## Future directions

The recent establishment and use of interactive platforms based on the internet websites such as LiverTox and HepaTox represent great progress in the field of DILI research [10, 11]. These websites provide important information about drug hepatotoxicity, terminologies, diagnostic tools, latest information, and interactive systems for case reports, management, and follow-up of DILI. Therefore, these platforms provide a convenient means for medical workers and the public to understand the scientific knowledge of DILI in time, maintain full vigilance to avoid the risks of DILI. These websites will also greatly promote basic and clinical research of DILI in the future.

The emphases of these platforms include:

1. To establish, improve, and use rationally a large database of DILI, as well as to establish a highly sensitive predicting and early warning system.
2. To launch more multicenter, prospective, randomized, controlled, rationally designed large-scale clinical studies, which have uniform terminology, diagnostic criteria, scientific designs, and better comparability, thus to promote deep understanding of the causes, natural history, clinical phenotypes, treatment strategy, and prognosis of DILI.
3. To apply “-omics” technology such as genomics, transcriptomics, proteomics, and metabolomics to assess the changes of genetic, immunological, molecular, biological, and biochemical events among individuals before and after the onset of DILI, followed by scientific statistical processing of the massive amount of information, thus to explore the occurring conditions of specific upstream events and nonspecific downstream events of signal transduction in DILI, analyze the regulatory patterns of those signal events, and clarify their internal correlations. This research will promote deep understanding of DILI pathogenesis, and help identify DILI biomarkers with better sensitivity and/or specificity, which will help identify the potential individuals who are susceptible, adaptable, or tolerable to specific drugs. These new biomarkers will improve the accuracy of prediction, early warning, prevention, diagnosis, and prognosis of DILI.
4. To understand the similarities and differences among liver injuries caused by various pathogenic factors.
5. To develop more accurate, practical, and convenient tools for the diagnosis of DILI based on the new-found diagnostic markers, thus break the limit of current methods for the diagnosis of DILI.
6. To investigate a more suitable biochemical criteria for the diagnosis of DILI in Chinese patients, and optimize the criteria for drug withdrawal after the occurrence of DILI.
7. To develop more effective drugs and establish more rational therapeutic patterns for the treatment of DILI.

Recommendations 14–16:

14. It is advisable to adopt different strategies and methods to achieve various goals for the management of DILI risk, including the identification of high-risk patients, drug withdrawal, reducing dosage, monitoring the changes of liver biochemical indexes at baseline and follow-ups, as well as weigh the balance of overall benefits and risks (1B)

15. Clinicians should carefully prescribe drugs according to the patients’ condition and drug indications and strictly obey the principles for drug compatibility and incompatibility. It is necessary to strengthen the public education and risk management of DILI, make the public and clinicians aware of the potential adverse reactions of TCM-NM-HP-DS, correct the mistaken concept that TCM-NM-HP-DS are always absolutely safe and nontoxic, and keep alert to the hepatotoxicity of folk remedies (1B)

16. The establishment, development, improvement, and use of interactive websites such as HepaTox and LiverTox will contribute to better understanding of DILI by medical staff and the public and should be fully used in the clinical practice and scientific research (1B)

## Abbreviations

ADR: Adverse drug reactions
AIH: Autoimmune hepatitis
AL-DILI: AIH-like DILI
AIHPAs: Anti-inflammatory and hepatoprotective agents
ALF; SALF: Acute liver failure; subacute liver failure
ALP: Alkaline phosphatase
ALT/AST: Alanine aminotransferase/aspartate aminotransferase
AMA: Anti-mitochondrial antibody
ANA: Anti-nuclear antibody
APAP: Acetaminophen
BCS: Budd–Chiari syndrome
CAMs: Complementary and alternative medications
CHB/CHC: Chronic hepatitis B/chronic hepatitis C
CK-18/CK-18FL/CK-18Fr: Cytokeratin 18/full length of CK-18/cytokeratin 18 fragment
CMV: Cytomegalovirus
CYP: Cytochrome P450
DILI/DILIN: Drug-induced liver injury/DILI network
DLST: Drug induced lymphocyte stimulation test
EBV: Epstein Barr virus
ERCP: Endoscopic retrograde cholangiopancreatography
ERSR/UPR: ER stress response/unfolded protein response
sFas: Soluble Fas
FasL; sFasL: Fas ligand; soluble FasL
GGT: Gamma-glutamyl transpeptidase
HAV/HBV/HCV/HEV: Hepatitis A virus/hepatitis B virus/hepatitis C virus/hepatitis E virus
HDS: Herbs and dietary supplements
HIV: Human immunodeficiency virus
HLA: Human leukocyte antigen
HMGB1: High mobility group box B1
IBD: Inflammatory bowel disease
IDILI/InDILI: Idiosyncratic DILI/intrinsic DILI
INR: International normalized ratio
IPH: Idiopathic portal hypertension
LKM: Liver and kidney microsomal antibody
MELD: Model for end stage liver disease
miRNA: MicroRNA
MRCP: Magnetic resonance cholangiopancreatography
MRI: Magnetic resonance imaging
NAC: *N*-Acetylcysteine
NAFLD: Non-alcoholic fatty liver disease
NRH: Nodular regenerative hyperplasia
NSAIDs: Non-steroidal antiinflammatory drugs
PBC: Primary biliary cirrhosis, primary biliary cholangitis
PH: Purpuric hepatis
PSC: Primary sclerosing cholangitis
RNA: Ribonucleic acid
RTR: Restorative tissue repair
RUCAM: The Roussel Uclaf causality assessment method
SEOP: Structured expert opinion process
SMA: Anti-smooth muscle antibody
SOS/VOD: Sinusoidal obstruction syndrome/hepatic veno-occlusive disease
TBil: Total bilirubin
TCM-NM-HP-DS: Traditional Chinese medicine, natural medicine, health products and dietary supplements
TCMp: TCM preparations ready for convenient clinical application and usually with the form of capsules, pills or tablets
TNF/sTNF: Tumor necrosis factors/soluble TNF
TNFR/sTNFR: Tumor necrosis factor receptors/soluble TNFR
TRAIL/sTRAIL: TNF-related apoptosis-inducing ligand/soluble TRAIL
UDCA: Ursodeoxycholic acid
ULN: Upper limit of normal
VBDS: Vanishing bile duct syndrome
